# Stimulus Feature-Specific Control of Layer 2/3 Subthreshold Whisker Responses by Layer 4 in the Mouse Primary Somatosensory Cortex

**DOI:** 10.1093/cercor/bhab297

**Published:** 2021-08-27

**Authors:** Stefano Varani, Dania Vecchia, Stefano Zucca, Angelo Forli, Tommaso Fellin

**Affiliations:** Optical Approaches to Brain Function Laboratory, Istituto Italiano di Tecnologia, 16163 Genova, Italy; Optical Approaches to Brain Function Laboratory, Istituto Italiano di Tecnologia, 16163 Genova, Italy; Optical Approaches to Brain Function Laboratory, Istituto Italiano di Tecnologia, 16163 Genova, Italy; Optical Approaches to Brain Function Laboratory, Istituto Italiano di Tecnologia, 16163 Genova, Italy; Optical Approaches to Brain Function Laboratory, Istituto Italiano di Tecnologia, 16163 Genova, Italy

**Keywords:** barrel cortex, cortical layers, direction selectivity, patch-clamp

## Abstract

In the barrel field of the rodent primary somatosensory cortex (S1bf), excitatory cells in layer 2/3 (L2/3) display sparse firing but reliable subthreshold response during whisker stimulation. Subthreshold responses encode specific features of the sensory stimulus, for example, the direction of whisker deflection. According to the canonical model for the flow of sensory information across cortical layers, activity in L2/3 is driven by layer 4 (L4). However, L2/3 cells receive excitatory inputs from other regions, raising the possibility that L4 partially drives L2/3 during whisker stimulation. To test this hypothesis, we combined patch-clamp recordings from L2/3 pyramidal neurons in S1bf with selective optogenetic inhibition of L4 during passive whisker stimulation in both anesthetized and awake head-restrained mice. We found that L4 optogenetic inhibition did not abolish the subthreshold whisker-evoked response nor it affected spontaneous membrane potential fluctuations of L2/3 neurons. However, L4 optogenetic inhibition decreased L2/3 subthreshold responses to whisker deflections in the preferred direction, and it increased L2/3 responses to stimuli in the nonpreferred direction, leading to a change in the direction tuning. Our results contribute to reveal the circuit mechanisms underlying the processing of sensory information in the rodent S1bf.

## Introduction

Rodents use whiskers to sense the world around them (Carvell and Simons 1990; Diamond et al. 2008; O'Connor, Clack, et al. 2010a; Staiger and Petersen 2021). Specific features of the physical interaction between whiskers and objects, for example the direction of whisker deflection, generate complex electrical signals in sensory neurons (Zucker and Welker 1969; Lichtenstein et al. 1990). These signals are relayed to primary sensory areas in the brain and they are believed to contribute to the computation of object location and shape (Diamond et al. 2008; Staiger and Petersen 2021). To understand how rodents use these specific features to detect and discriminate objects, it is thus essential to identify the cellular mechanisms responsible for their processing in the primary somatosensory cortex (S1), a key cortical region involved in the integration of whisker-related information.

In S1bf, the information about the angular direction of whisker deflection is inherited from the ventral posteromedial nucleus (VPM) of the thalamus (Bruno et al. 2003) at the level of L4, the principal target of VPM thalamocortical projections (Chmielowska et al. 1989; Staiger et al. 1996; Feldmeyer 2012). Axonal projections of L4 principal cells contact neighboring L4 cells (Feldmeyer et al. 1999; Lübke et al. 2000) and superficial L2/3 neurons (Lübke et al. 2000; Feldmeyer et al. 2002; Lubke et al. 2003), providing strong feedforward excitation, which, according to the canonical model, drives these superficial layers (Douglas and Martin 2004). Despite the inputs from L4 excitatory cells, the strength of the directional tuning in suprathreshold response of L2/3 excitatory cells is debated (Simons and Carvell 1989; Bruno and Simons 2002; Minnery et al. 2003; Lee 2004; Andermann and Moore 2006; Kerr et al. 2007; Kremer et al. 2011; Bale and Maravall 2018; Kwon et al. 2018). These results are in agreement with the observation that besides L4 inputs, L2/3 principal neurons receive additional input fibers (Feldmeyer 2012; Staiger and Petersen 2021) including horizontal connection from neighboring L2/3 cells (Bureau et al. 2006; Feldmeyer et al. 2006; Lefort et al. 2009; Adesnik and Scanziani 2010), long-range inputs from other cortical areas (Petreanu et al. 2007; Petreanu et al. 2009; Aronoff et al. 2010; Mao et al. 2011; Banerjee et al. 2020), and ascending fibers from thalamic nuclei (Petreanu et al. 2009; Meyer et al. 2010; Oberlaender et al. 2012; Audette et al. 2018).

L2/3 pyramidal cells are characterized by sparse firing activity and unreliable suprathreshold sensory-evoked responses, with a small fraction of highly active neurons accounting for the majority of the stimulus-evoked spikes (de Kock et al. 2007; Kerr et al. 2007; O'Connor, Peron, et al. 2010b; Crochet et al. 2011; Barth and Poulet 2012; Petersen and Crochet 2013; Peron et al. 2015). In contrast with the sparse sensory-evoked firing activity, whole-cell membrane potential recordings from L2/3 excitatory neurons reveal large-amplitude and reliable sensory-evoked subthreshold responses (Brecht et al. 2003; Petersen et al. 2003; Crochet and Petersen 2006; Crochet et al. 2011; Vecchia et al. 2020). Recording the subthreshold membrane potential dynamics of L2/3 cells thus provides the opportunity to study the integration of multiple inputs with reliable signals. In anesthetized rats, L2/3 pyramidal neurons display direction selectivity at the subthreshold level (Brecht et al. 2003; Ramirez et al. 2014), but whether similar properties are present in awake animals is currently unknown. Moreover, whether direction selectivity of subthreshold response in L2/3 is mainly inherited from L4, as posited by the canonical model, or whether it results from the integration of multiple inputs to L2/3 is currently unclear.

Here, we performed in vivo patch-clamp recordings in L2/3 pyramidal cells and 2-photon-guided juxtasomal recordings during whisker stimulation in both anesthetized and head-restrained awake mice in combination with cell-specific optogenetic inhibition of L4 to address these questions.

## Materials and Methods

### Animals

Experimental protocols involving animals were approved by the IIT Animal Health Regulatory Committee and the National Council on Animal Care of the Italian Ministry of Health (authorization #34/2015-PR and 125/2012-B). Experiments were conducted according to National legislation and to the guidelines of the European Communities Council Directive. The mouse lines Scnn-Cre (B6;C3-Tg(Scnn1a-cre)3Aibs/J, stock #009613) and PV-Cre (B6;129P2-Pvalb^tm1(cre)Arbr^/J, stock #008069) were crossed with the TdTomato (TdTom) reporter line (B6;129S6-Gt(ROSA)26Sor^tm14(CAG-TdTomato)Hze^/J, stock #007908) and were purchased from the Jackson Laboratory. Experiments were performed in juvenile mice (4–10 weeks old, either sex) housed in singled ventilated cages (maximum 5 animals per cage, divided by sex) and maintained under a 12:12 light–dark cycle with ad libitum access to food and water.

### Viral Injections

Scnn-Cre × TdTom mice were injected with adeno-associated viruses (AAVs) AAV1.EF1a.DIO.eNpHR3.0-eYFP.WPRE.hGH (Halo; Addgene viral prep #26966-AAV1; RRID: Addgene_26 966, titer 2-3E13 gc/mL, injected with a 1:1 dilution ratio) or AAV1. CAG.Flex.eGFP.WPRE.bGH (eGFP; Addgene viral prep #51502-AAV1; RRID: Addgene_51 502, titer 9.2E12 gc/mL, injected with no dilution). PV-Cre × TdTom mice were injected with AAV1. EF1.dflox.hChR2(H134R)-mCherry.WPRE.hGH (Chr2; Addgene viral prep #20297-AAV1; RRID: Addgene_20 297, titer 4.5E13 gc/mL, injected with no dilution). All stereotaxic injections were performed between postnatal day 0 (P0) and P2 as described in De Stasi et al. (2016), and AAVs were purchased from the University of Pennsylvania Viral Vector Core. Briefly, each pup was deeply anesthetized by hypothermia, immobilized in a customized stereotaxic apparatus and kept at approximately 4°C. A small skin incision was performed along midline to expose the skull, and a glass micropipette was lowered at stereotaxic coordinates of (with respect to bregma): 0 mm caudal, 1.5 mm lateral, and 0.25 mm depth. About 200–300 nL of virus suspension were injected slowly and the micropipette was held in place for 1 min to prevent spilling of the virus during the retraction. The micropipette was then gently removed and the skin was sutured. The animal was warmed under a heating lamp to recover normal body temperature and movements and finally returned to its home cage.

### Surgery: Anesthetized Mice

Mice were anesthetized with an intraperitoneal injection of urethane (16.5%, 1.65 g/kg). The body temperature was constantly monitored with a rectal probe and kept at 36.5–37°C with a heating pad. The animals were maintained in a state of deep anesthesia for the entire duration of the surgery or until the end of the experiment. Depth of anesthesia was monitored by controlling the respiration rate, hear-beat frequency, eyelid reflex, reaction to tail and toes pinching, absence of vibrissae movements. In some experiments oxygen saturation was controlled by a pulse oximeter (MouseOx, Starr Life Sciences Corp.). Lidocaine solution (2%) was locally injected under the skin in the area of the surgery. The skin was cut to expose the skull, and the area above the barrel field of the primary somatosensory cortex (S1bf) was thinned to allow intrinsic optical imaging (IOI) in order to identify the cortical region where to perform the craniotomy. IOI was performed with a customized setup similarly to Zucca et al. (2019) and Vecchia et al. (2020). Before acquiring images for IOI, all but one whisker (usually C2) were trimmed in the contralateral whisker pad with respect to the virus injection site. The spared whisker was put inside a glass capillary tube, which was glued to a piezoelectric bender actuator (Physik Instrumente). The skull was illuminated with red light (wavelength: 630 ± 10 nm) and time series images were acquired with a camera (Hamamatsu). The whisker was stimulated at 18 Hz for 1.1 s at intervals of 20 s for a total of 40 trials. Camera frames were averaged over trials and a custom MATLAB (version 2017b, The MathWorks, Inc.) script based on Harrison et al. (2009) was used to analyze images. The region characterized by decreased reflectance relative to baseline identified the principal barrel column corresponding to the stimulated spared whisker. Subsequently an image of vasculature with green light (wavelength: 546 ± 10 nm) was acquired as spatial reference. A small craniotomy (<1 mm^2^) was performed over the S1bf area identified by IOI without removing the dura and normal HEPES-buffered artificial cerebrospinal fluid (aCSF) was used to keep the brain surface moist through the whole duration of the experiment.

### Surgery: Awake Head-Fixed Mice

For head-restrained experiment in awake animals (Figs 4 and 6; Supplementary Figs 3 and 6), a custom head plate was fixed to the skull at P28–30 to achieve stable head fixation. The mouse was anesthetized with isoflurane (2%/O_2_ 1 L/min), the body temperature was kept at approximately 36.5°C with a heating pad, and an ophthalmic solution was frequently applied to keep the eyes humid. The scalp was disinfected with Betadine and was cut to expose the skull. A total of 2% lidocaine solution was injected under the skin before surgical incision. A screw was implanted on the hemisphere contralateral to the injection site to improve stability, and the head plate was fixed on the skull with dental cement posterior to S1bf, avoiding the area selected for the craniotomy. Kwik-Cast silicone elastomer (World Precision Instruments) was applied over the exposed bone and antibiotic (BAYTRIL) was administered via intraperitoneal injection to prevent infection. After 2–3 days of recovery from the surgery, animals were habituated to head-restrain sitting in a plastic tube during yellow light illumination (Figs 4 and 6; Supplementary Figs 3 and 6) and to piezoelectric stimulation of randomly chosen whiskers (Fig. 6 and Supplementary Fig. 6) for a minimum of 10 days similarly to (Gentet et al. 2010). The duration of head-restrained sessions gradually increased with days. Electrophysiological recordings were performed after the animal sat quietly in the recording environment. On the day of the experiment, the animal was anesthetized with isoflurane (2%/O_2_ 1 L/min) and a craniotomy was opened in the region identified by IOI, as described in the previous section. The recording session started after at least 30 min from the recovery from anesthesia.

### Optical and Whisker Stimulation

Yellow light (λ = 594 nm) and blue light (λ = 488 nm) illumination (duration: 1 s) were performed with a continuous-wave, solid-state laser source (Cobolt). In all inhibitory optogenetics experiments with exception of Supplementary Figure 2 the 1-s-long light stimulus ended with a ramp-like reduction in light power (ramp duration: 100 ms) to minimize the neural spiking rebound (Mahn et al. 2016). Yellow light power was controlled with an acousto-optic modulator (R23080-3-LDT, Gooch & Housego PLC), and both yellow and blue lights were delivered to the brain through an optical fiber cable with a diameter of 940 μm and numerical aperture of 0.22 (QMMJ-3XF-UVVIS-940/1000-3-3, AMS Technologies). Yellow and blue lights were presented at intensity of ≤30 and ≤18 mW, respectively. The intensity of the light was measured with a digital optical power meter (Thorlabs) placed proximally to the fiber tip. The optical fiber cable was placed approximately 1 mm above the craniotomy. Before the experiment, the whisker was trimmed to a length of approximately 1.5 cm. Hold and release passive single whisker stimulations (duration: 500 ms; deflection amplitude: ~2 mm) were performed placing the targeted whisker inside a glass pipette attached to a piezoelectric bender actuator (Physik Instrumente). When whisker stimulation was coupled with optogenetic manipulation, light delivery started 100 ms ahead of the whisker stimulus onset. For the experiments in Figures 2 and 3 and Supplementary Figures 2 and 4 whiskers were deflected in one direction, mostly rostro-caudally (0° with respect to the horizontal alignment of the whisker rows). For the experiments shown in Figures 5 and 6 and Supplementary Figures 5 and 6, the piezoelectric actuator was connected to either a manual metric rotation stage (MSRP01/M, Thorlabs) or a custom-made motorized rotation stage mounted on a flexible holder, allowing the deflection of the whisker at different angular direction. The whisker was pseudorandomly deflected at angles of 0°, 45°, 90°, and 315° with respect to the horizontal alignment of the whisker rows.

### Patch-Clamp Recordings

Glass pipettes (Hilgenberg) were filled with internal solution containing in mM: K-gluconate 140, MgCl_2_ 1, NaCl 8, Na_2_ATP 2, Na_3_GTP 0.5, HEPES 10, Tris-phosphocreatine 10 to pH 7.2 with KOH (all by Sigma-Aldrich). For electrophysiological recordings of L2/3 pyramidal cells, pipette with resistance of 3–6 MΩ were used, whereas for L4 recordings the pipette resistance was in the range of 7–14 MΩ. Cells depth within the tissue was inferred from the position of the glass pipette with respect to the pial surface. The range of depths was 110–380 μm for L2/3 cells and 410–500 for L4 cells. For experiments in Figures 3 and 4 and Supplementary Figures 2–4, 20–30 consecutive acquisitions (trials, acquisition duration: 4–8 s) were performed for each experimental condition and for experiments in Figures 5 and 6 and Supplementary Figures 5 and 6, for each stimulus direction. For experiments in Figure 2, 12–30 trials, for each experimental condition, were acquired. Data were collected through a Multiclamp 700B amplifier, sampled at 50 kHz, and filtered at 10 kHz by a Digidata 1440 acquisition system (Axon Instruments).

### Two-Photon Targeted Juxtasomal Recordings

Surgery was performed as described above, with the exception that the dura was removed. The imaging setup was composed of: 1) a Chameleon Discovery pulsed laser source (Coherent Italy) tuned at wavelength 920, 980, or 1020 (the excitation power was measured at the focal plane of the objective with a digital optical power meter (Thorlabs) and was set between 30 and 80 mW); 2) a laser scanning Ultima II scanhead (Bruker Italy); 3) a ×40 0.80 NA objective (Olympus) or ×16 0.80 NA objective (Nikon); 4) 2 photomultiplier tubes for both green and red fluorescence collection (Hamamatsu). Juxtasomal recordings were performed as described in Forli et al. (2018). Briefly, 5–7 MΩ glass pipettes (Hilgenberg) were filled with aCSF solution containing Alexa Fluor 488 (concentration: 20 μM; #A10436, Thermo Fisher Scientific). TdTom^+^ neurons in L4 were targeted while monitoring fluorescence of the glass pipette and applying a slight positive pressure to prevent pipette clogging. When the electrode was in proximity of a targeted cell the positive pressure was released and a negative pressure was used to achieve the juxtasomal configuration. In all experiments, 30 consecutive trials (acquisition duration: 4–8 s) were performed for each experimental condition. Electrical signals were amplified and digitized as for the patch-clamp recordings described above.

### Immunohistochemistry and Confocal Image Acquisition

**Figure 1:**
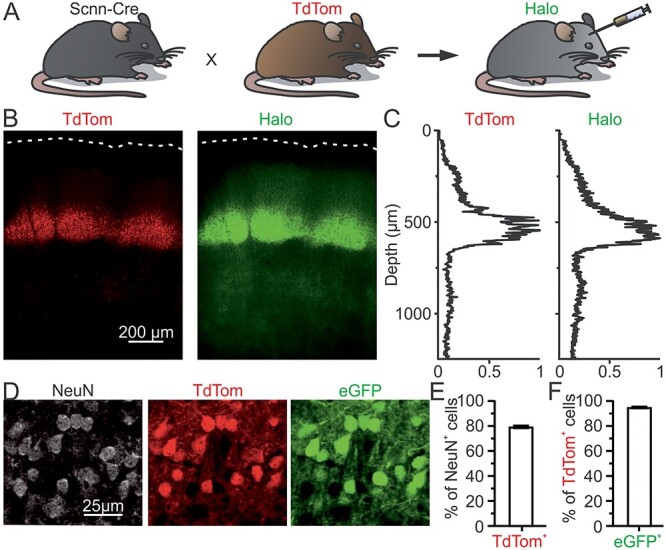
Selective expression of Halo in L4 of S1bf. (*A*) Schematic representation of the transgenic mouse model used in this study. Bigenic Scnn-Cre × TdTom mice were injected with AAVs carrying the conditional Halo-eYFP construct. (*B*) Confocal images of a coronal section showing TdTom (left) and Halo (right) expression in the S1bf. White dotted lines highlight the cortical surface. (*C*) Normalized fluorescence intensity as a function of cortical depth for the images shown in *B*. (*D*) Confocal images of a coronal section of a Scnn-Cre × TdTom mouse injected with AAVs carrying the conditional eGFP construct. NeuN staining (left), TdTom fluorescence (center), and eGFP fluorescence (right) in L4 neurons are shown. (*E*) Percentage of NeuN^+^ expressing TdTom (TdTom^+^). *n* = 36 fields of view from 3 animals, error bar represents SEM. (*F*) Percentage of TdTom^+^ cells expressing eGFP (eGFP^+^). *n* = 36 fields of view from 3 animals, the error bar represents the SEM.

Scnn-Cre × TdTom mice (4–6 weeks old) injected with AAV-transducing eGFP were perfused transcardially with 0.01 M PBS (pH 7.4) and then with 4% paraformaldehyde in phosphate-buffered saline (PBS). The brains were postfixed overnight at 4°C and then transferred to in a cryoprotectant solution (30% sucrose in PBS). Fixed brains were cut to obtain coronal sections of 40 μm. The sections were incubated overnight in a solution containing 0.4% mouse anti-NeuN (RRID: AB_2298772; Millipore MAB377), 0.01 M PBS (pH 7.4), 0.5% Triton X-100, and 1% normal serum of the same species as the secondary antibody. The next day, the sections were incubated at room temperature in goat anti-mouse Alexa 647 (1:800, RRID: AB_141725, Molecular Probes [Invitrogen]) secondary antibodies with 0.01 M PBS (pH 7.4) in 0.5% Triton X-100 for 1 hour. The sections were finally mounted on a glass slide with 1,4 diazobicyclo-(2,2,2)octane (DABCO) mounting medium and coverslipped. For the cell count analysis shown in Figure 1, confocal z-stacks (512×512 pixels, 2 μm z steps, 40x magnification) of the S1bf were acquired using a Leica SP5 inverted confocal microscope (Leica Microsystems). Four consecutive sections were analyzed in each mouse, imaging the whole thickness of the sections. The cells were counted manually using ImageJ (version 1.50f, Fiji) with the grid and cell counter plugins (square area, 5000 μm^2^). NeuN^+^, TdTom^+^, and eGFP^+^ cells were counted in 3 randomly chosen squares placed inside a L4 barrel. Cells that crossed the upper and right borders of the grid were included, whereas those that crossed the lower and left borders were excluded from the counts. Data were normalized to the total number of NeuN^+^ cells and averaged across the sections for each animal. The mean values obtained across sections from 1 animal were averaged across animals. For the cell count analysis shown in Supplementary Figure 1 confocal z-stacks (1024 × 1024 pixels, 5-μm z steps, ×20 magnification) of the S1bf were acquired using a Leica SP5 inverted confocal microscope (Leica Microsystems). A total of 23 sections from 2 animals were analyzed, for a total of 44 barrels. For each barrel, the borders of L2/3 were manually assigned by 2 operators looking at the acquired z stack median projection (for both the NeuN and eGFP signals) based on cell density, cell size, and tissue anatomy. eGFP^+^ cells in the L2/3 were manually counted using ImageJ, and their density in the investigated volume was averaged across samples.

### Data Analysis

**Figure 2:**
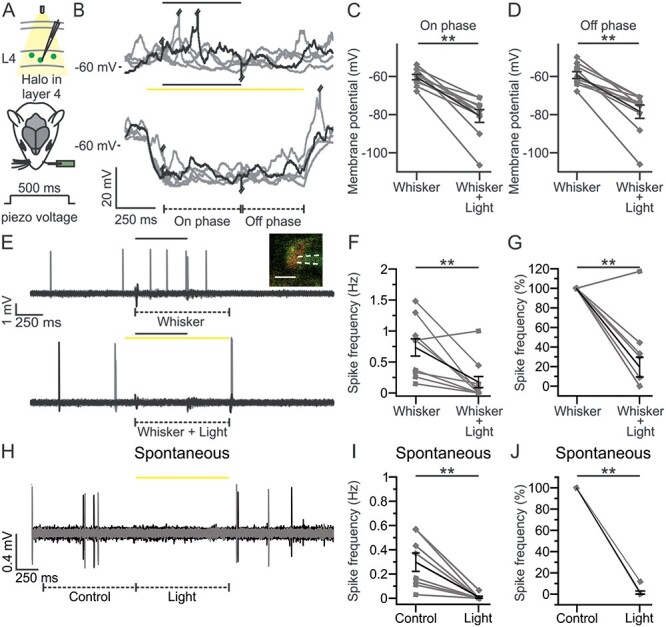
Halo-mediated inhibition of L4 excitatory neurons during whisker stimulation. (*A*) Schematic representation of the experimental configuration for electrophysiological recordings in L4 excitatory neurons expressing Halo (green cells) in S1bf of anesthetized mice. A single whisker contralateral to the injected hemisphere was spared and stimulated using a glass capillary connected to a piezo actuator (whisker deflection duration: 500 ms). Yellow light was delivered to the barrel cortex through a fiber optic (duration of optogenetic stimulus: 1 s). In this as well in other figures, opsin-positive cells are colored, whereas opsin-negative cells are indicated in gray. (*B*) Five representative traces of membrane potential responses recorded in the whole-cell configuration from a L4 Halo-positive neuron during whisker stimulation in the absence (top) or presence of optogenetic inhibition of L4 (bottom) in anesthetized mice. In this as well as in other figures, the black and the yellow bars represent the timing of the whisker stimulus and of the optogenetic illumination, respectively. Piezo artifacts and APs were truncated when necessary for presentation purposes. Whisker responses were divided into 2 phases: on phase (from 10 to 500 ms after the whisker stimulation onset) and off phase (from 10 to 400 ms after the end of the deflection). (*C*) Membrane potential of Halo-positive cells during the on phase of the whisker stimulation in the absence (Whisker) or presence of yellow light illumination (Whisker + Light). *n* = 10 cells from 8 animals; Wilcoxon signed rank test, *P* = 2E-3. In this as well as in other figures, the values from individual cells are shown in gray, the average of all cells in black. Error bars indicate SEM. (*D*) Same as in *C*, but during the off phase of the whisker response. *n* = 10 cells from 8 animals; Wilcoxon signed rank test, *P* = 2E-3. (*E*) Ten representative traces from a 2-photon guided juxtasomal recordings from a TdTom^+^ L4 cell coexpressing Halo during whisker stimulation alone (top) or during combined optogenetic inhibition of L4 (bottom) in anesthetized mice. The dashed lines below the traces indicate time windows of 900 ms during whisker stimulation (Whisker) or during combined sensory stimulation and yellow light stimulation (Whisker + Light). Inset: 2-photon image showing the recording pipette and the recorded cell expressing Halo (green) and TdTom (red). Scale bar: 10 μm. White dotted lines indicate the glass pipette. (*F*) Spike frequency (Hz) of L4 TdTom^+^ neurons in the Whisker and Whisker + Light time windows. *n* = 12 cells from 10 animals; Wilcoxon signed rank test, *P* = 2E-3. In one out of 12 cells yellow light did not decrease the spike frequency suggesting that only 11 out of the 12 TdTom^+^ cells were also Halo^+^ cells (92%, in agreement with what shown in Fig. 1*F*). The sensory-evoked spike rate in the 11 light-responsive cells was reduced by yellow light stimulation (spike frequency: 0.72 ± 0.15 Hz [Whisker] versus 0.10 ± 0.05 Hz [Whisker + Light]; Wilcoxon signed rank test, *P* = 1E-3) to 11%. (*G*) Same as in *F* but normalized to the spike frequency during whisker stimulation (Whisker). *n* = 12 cells from 10 animals, Wilcoxon signed rank test, *P* = 3E-3. (*H*) Same as in *E* during spontaneous activity (Control) or during optogenetic inhibition of L4 (Light). (*I*) Same as in *F* for TdTom^+^ cells recorded under control conditions and during optogenetic inhibition of L4. *n* = 8 cells from 3 animals; Wilcoxon signed rank test, *P* = 8E-3. (*J*) Same as in *I*, but normalized to the spike frequency under control conditions (Control). *n* = 8 cells from 3 animals, Wilcoxon signed rank test, *P* = 8E-3. In this as well in the other figures: ^*^P ≤ 0.05; ^*^^*^P ≤ 0.01; ^*^^*^^*^P ≤ 0.001.

**Figure 3:**
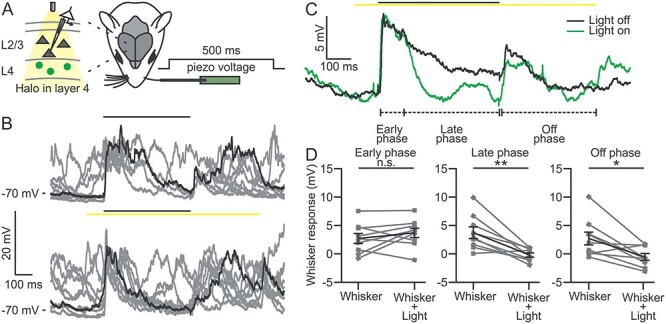
Optogenetic inhibition of L4 neurons decreases but does not suppress the subthreshold response to whisker deflection in L2/3 pyramidal cells. (*A*) Schematic representation of the experimental configuration for current-clamp recordings in L2/3 pyramidal neurons during single whisker stimulation and optogenetic inhibition of L4 in anesthetized mice. Whisker and optogenetic stimulation were performed as described in Figure 2*A*. (*B*) Ten representative traces showing the membrane potential responses of a L2/3 pyramidal neuron during whisker stimulation in the absence (top) and presence (bottom) of optogenetic inhibition of L4. (*C*) Average membrane potential response across 30 trials in the absence (black) and presence (green) of optogenetic inhibition of L4 for the cell shown in *B*. In this as well as in the other figures: early phase, 10–100 ms from the onset of whisker stimulation; late phase, 100–500 ms from the onset of whisker stimulation; off phase, 10–400 ms from the end of whisker deflection. (*D*) Whisker-evoked responses in L2/3 pyramidal neurons during whisker stimulation in the absence (Whisker) and presence of optogenetic inhibition of L4 (Whisker + Light) for the early (left), late (center), and off (right) temporal windows. *n* = 9 cells from 4 animals; paired Student’s *t*-test, *P* = 0.27 for early phase; *P* = 3E-3 for late phase; *P* = 0.013 for off phase.

In intracellular recordings shown in Figure 2, neurons were considered positive for the inhibitory opsin Halo (Halo-positive) if they presented on average a net hyperpolarization of their membrane potential (>3 times the standard deviation [SD] of the control membrane potential) upon yellow light illumination. For the juxtasomal recordings, we collected data only from neurons that showed red fluorescence (TdTom^+^). For data in Figures 2 and 3 and Supplementary Figures 2 and 4, Clampfit 10.2 (Molecular Device) was used for the quantification of the number of spikes, the mean membrane potential, and the whisker-evoked depolarization. In Figure 2*B*–*D* the on phase was defined as the time window between 10 and 500 ms after whisker stimulation onset and the off phase as the time window between 10 ms and 400 ms after the end of whisker deflection. In Figure 2*E*–*J* the mean values of spike rate were computed averaging individual spikes across trials and then across neurons. In Figure 2*E*–*G* spikes were quantified in a time window of 900 ms, beginning at the onset of the whisker stimulus and ending at the offset of the light illumination. In Figure 2*H*–*J*, a 1-s-long time window was considered for quantification (from the start to the end of the light illumination [Light] or starting 1 s before the onset of the optogenetic stimulation [Control]). For experiments in anesthetized mice reported in Figures 3 and 5 and Supplementary Figures 2–5, we divided the response to the whisker stimulation in 3 phases: early phase (from 10 to 100 ms after the whisker stimulation onset), late phase (from 100 to 500 ms after the whisker stimulation onset), and off phase (from 10 to 400 ms after the end of whisker deflection). The first 10 ms after the start and the end of the whisker stimulation were excluded from the analysis to avoid artifacts due to the activation of the piezoelectric actuator. The whisker response was defined as the difference between the mean membrane potential in each phase (early, late, and off) and the mean of the membrane potential recorded in the 25-ms-long time window (baseline) preceding the whisker stimulus onset (prestimulus phase). The same baseline was used to quantify the sensory response in the early, late, and off phases.

For the analysis of the spontaneous activity reported in Supplementary Figure 3, recordings that showed oscillatory dynamics characterized by a bimodal membrane potential distribution were considered as synchronized. Specifically, we adapted a method similar to Mukovski et al. (2007) for intracellular recordings. For each trial, we calculated the ratio between the power at low frequency (0.5–4 Hz) and at higher frequency (4–50 Hz) and we selected trials with a ratio value >50 and with mean membrane potential of the whole trial ≤50 mV. In the same recordings, desynchronized periods were defined as trials with value <30 of the ratio between the power at low and higher frequency. For each condition (synchronized and desynchronized), classified trials were visually validated by 2 expert operators and cells with <10 trials meeting the criteria described above were excluded. For each cell, the mean and the SD of the membrane potential in a 1 s-long time window during light illumination (Light) and in a 1 s-long time window right before the illumination (Control) were computed in all the selected trials.

**Figure 4:**
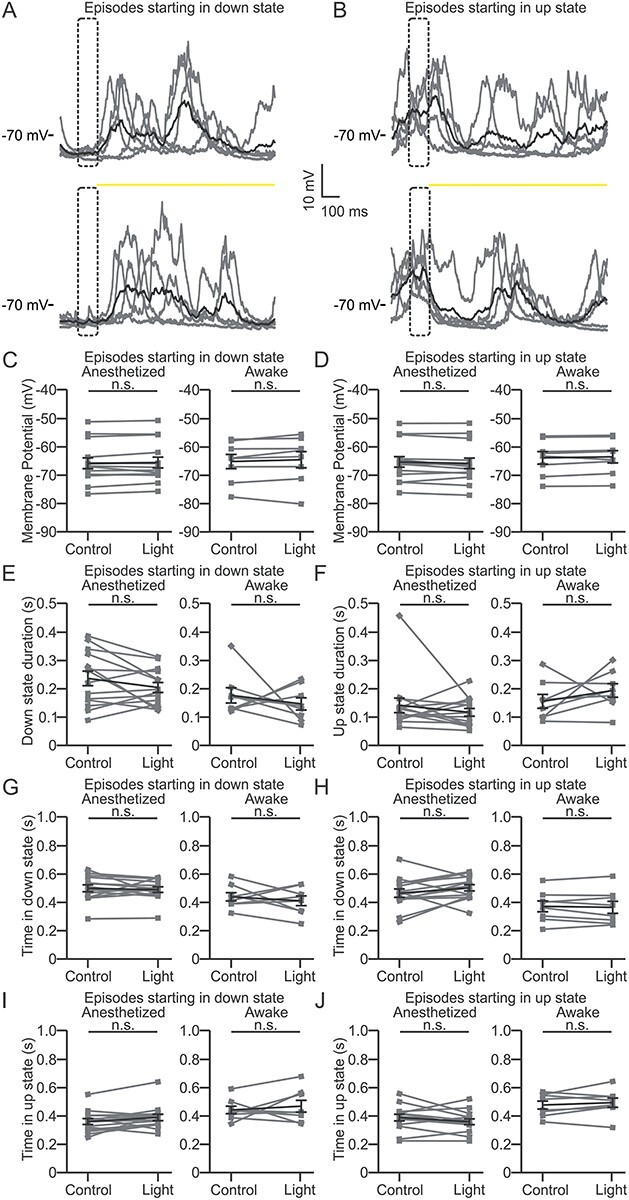
Optogenetic inhibition of L4 does not affect spontaneous activity recorded in L2/3 pyramidal cells. (*A*) Representative current-clamp recordings showing the membrane potential of a L2/3 pyramidal cell during spontaneous activity in an anesthetized animal. Episodes were selected based on the presence of a down state during a 100-ms time window (prewindow, dotted box) preceding either a control period (top) or the yellow light stimulation (bottom). Yellow light stimulation is indicated by the yellow bar. Five trials (gray line) and their average (black line) are shown. (*B*) Same as in *A*, but for traces showing an up state during the prewindow. (*C*) Left: average membrane potential for episodes starting with a down state during the control period (Control) and optogenetic inhibition of L4 (Light) in anesthetized animals. *n* = 14 cells in 6 animals. Right: same as in left for awake head-fixed animals. *n* = 8 cells in 7 animals. Paired Student’s *t*-test: *P* = 0.33 and 0.29 for anesthetized and awake condition, respectively. (*D*) Left: average membrane potential for episodes starting with an up state during the control period (Control) and yellow light stimulation (Light) in anesthetized animals*. n* = 14 cells from 6 animals. Right: same as in left for awake animals. *n* = 8 cells from 7 animals. Paired Student’s *t*-test: *P* = 0.11 and 0.24 for anesthetized and awake condition, respectively. (*E*) Same as in *C* for length of the initial down state for episodes starting with a down state: paired Student’s *t*-test: *P* = 0.14 and Wilcoxon paired signed rank test *P* = 0.74 for anesthetized and awake condition, respectively. (*F*) Same as in *D* for length of the initial up state for episodes starting with an up state: Wilcoxon paired signed rank test *P* = 0.46 and paired Student’s *t*-test: *P* = 0.31 for anesthetized and awake condition, respectively. (*G*) Same as in *C* for total time spent in down state (time in down state) for episodes starting with a down state: Wilcoxon paired signed rank test *P* = 0.58 and paired Student’s *t*-test: *P* = 0.50 for anesthetized and awake condition, respectively. (*H*) Same as in *D* for the total time spent in down state (time in down state) for episodes starting with an up state: paired Student’s *t*-test: *P* = 0.19 and *P* = 0.60 for anesthetized and awake condition, respectively. (*I*) Same as in *C* for the total time spent in up state (time in up state) for episodes starting with a down state: Wilcoxon paired signed rank test *P* = 0.10 and paired Student’s *t*-test: *P* = 0.53 for anesthetized and awake condition, respectively. (*J*) Same as in *D* for the total time spent in up state (time in up state) for episodes starting with an up state: paired Student’s *t*-test: *P* = 0.13 and 0.46 for anesthetized and awake condition, respectively.

For the up and down state analysis shown in Figure 4, only synchronized trials were considered. In selected trials, we adopted a simplified version of the method described in Zucca et al. (2017) and Zucca et al. (2019)) to determine 2 thresholds used for the identification of putative up- and down-like states. Briefly, trials were down-sampled to 10 kHz and smoothed with a 50-ms running frame linear filter (Mukovski et al. 2007). The resulting trace was low pass filtered in the 0.1–20 Hz range and the top first percentile was excluded to obtain the evidence variable *S(t).* We fitted *S(t)* with a mixture of 2 Gaussians using an expectation maximization algorithm (Saleem et al. 2010; Zucca et al. 2017). Means and variances of the Gaussians were identified as μUP, μDOWN, and σUP, σDOWN, respectively. We performed the same preprocessing to obtain *S(t)* on the whole recording and time samples corresponding to *S(t)* > μUP − σUP were assigned to up states, whereas samples corresponding to *S(t)* < μDOWN + σDOWN were assigned to down states. The remaining time samples with *S(t)* values in between the 2 thresholds were defined as indeterminate state. According to Zucca et al. (2017) and Zucca et al. (2019) the minimum state duration and the minimum interstate interval were set equal to 100 and 50 ms, respectively. Recordings were excluded from the analysis if the thresholds set for the 2 states were overlapping. We defined light episodes (duration: 1 s) as the periods under light illumination and control episodes (duration: 1 s) as the periods after 1.8 s from the end of the light illumination. We classified episodes as occurring during an ongoing up or down state based on the presence of an identified putative up- or down-like states (see above) in a short time window (duration: 100 ms) before the start of each period. For analysis, we kept recordings with at least 5 episodes for each case (i.e., light in up state, light in down state, control in up state, and control in down state). The mean membrane potential was calculated from the start to the end of each control and light episode, respectively. The length of the starting state (either down or up state) was calculated from the start of each episode to the end of down or up state, respectively. The total time spent in either up or down state was calculated from the start to the end of each episode (duration: 1 s).

For intracellular recordings in Figures 2–4 and Supplementary Figures 2 and 4, cells with a mean resting membrane potential > −50 mV or a change in the resting membrane potential >20 mV during the course of the experiment were excluded from the analysis.

**Figure 5:**
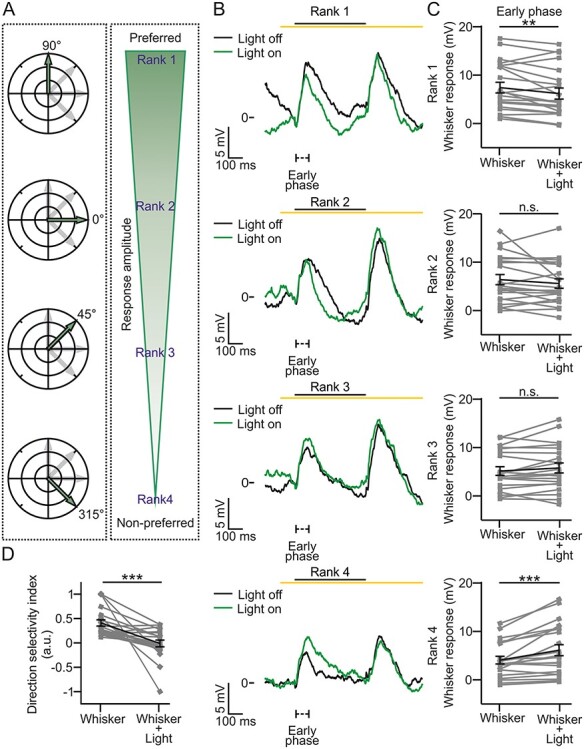
Stimulus direction-specific control of L2/3 whisker response by L4: anesthetized animals. (*A*) Left: for each neuron, a single whisker was stimulated in 4 different angular directions with respect to the rostral–caudal axis as shown in the polar plots. Right: based on the amplitude of the evoked response in the early phase of the control condition (Whisker), directions were ranked as follows (from top to bottom): preferred (1), second best preferred (2), third best preferred (3), and least preferred (4). (*B*) Traces showing the average membrane potential of a L2/3 neuron in the absence (black, Light on) and presence (green, Light off) of optogenetic inhibition of L4 during whisker stimulation in anesthetized mice for the different ranks (1–4, from top to bottom). (*C*) Amplitude of whisker-evoked responses for the different ranks for the early phase. *n* = 21 cells from 12 animals. Rank 1: Wilcoxon paired signed rank test, *P* = 0.0096. Rank 2: paired Student’s *t*-test, *P* = 0.18. Rank 3: paired Student’s *t*-test, *P* = 0.11. Rank 4: paired Student’s *t*-test, *P* = 6E-4. (*D*) Average DSI calculated in the absence (Whisker) or presence (Whisker + Light) of optogenetic inhibition of L4. *n* = 21 cells from 12 animals. Wilcoxon paired signed rank test, *P* = 7E-5.

**Figure 6:**
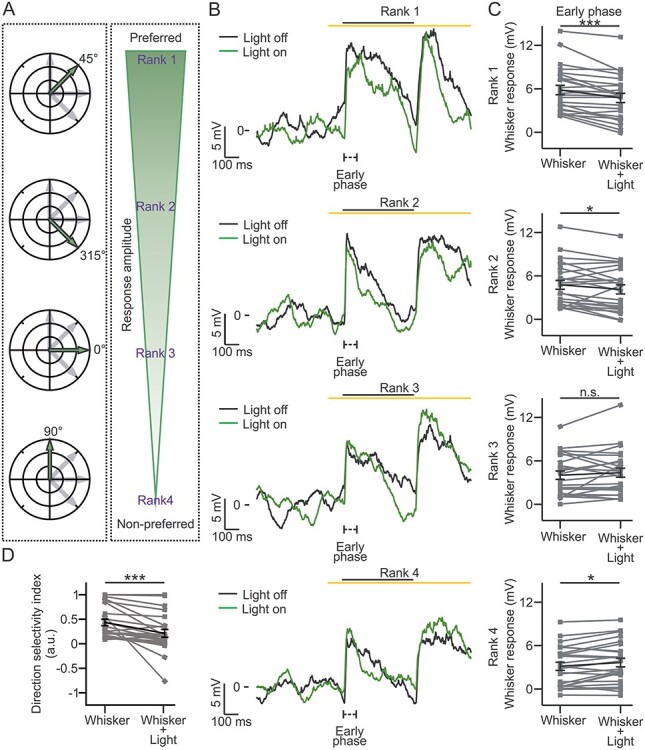
Stimulus direction-specific control of L2/3 whisker response by L4: awake animals. (*A*) Same as in Figure 5*A* for experiments in awake mice. (*B*, *C*) Same as in Figure 5*B*,*C* for recordings in awake animals. *n* = 24 cells from 11 animals. Rank 1: Wilcoxon paired signed rank test, *P* = 3E-4. Rank 2: Wilcoxon paired signed rank test, *P* = 0.016. Rank 3: Wilcoxon paired signed rank test, *P* = 0.55. Rank 4: paired Student’s *t*-test, *P* = 0.013. (*D*) Same as in Figure 5*D* for recordings in awake mice. *n* = 24 cells from 11 animals. Wilcoxon paired signed rank test, *P* = 2E-5.

For the analysis of the whisker response during different stimulus directions in Figures 5 and 6 and Supplementary Figures 5 and 6, a custom Python 3.7 (https://www.python.org/) script was used and data were subsampled to 10 kHz. The mean of the prestimulus phase for anesthetized experiments in Figure 5 and Supplementary Figure 5 was calculated as described above, whereas for awake experiments displayed in Figure 6 and Supplementary Figure 6 it was calculated between −600 and −100 ms relative to the whisker stimulus onset. Early, late, and off phases and the whisker response were defined as described above.

For the analysis of the direction selectivity of L2/3 pyramidal cells (Figs 5 and 6 and Supplementary Figs 5 and 6), we ranked (from 1 to 4) the directions of the whisker deflection based on the amplitude of early phase of the deflection-induced response during whisker stimulation alone (Whisker). The ranking of the early phase of the whisker stimulation described above was used for the late and off phases of the whisker control condition (Whisker) and for all the 3 phases of the whisker stimulation combined with L4 optogenetic inhibition (Whisker + Light). We defined the direction selectivity of each cell in the early phase as:

$$ DSI=\frac{Rrank1- Rrank4}{\left(\left| Rrank1\right|+\left| Rrank4\right|\right)} $$

where *Rrank*1 was the response in the preferred direction and *Rrank*4 was the response in the least preferred direction. To be included in the analysis, recordings had to meet the following criteria: 1) action potential (AP), when present, had to reach a membrane potential ≥ −20 mV; 2) the membrane potential value of the lower fifth percentile was > −95 mV and < −55 mV; 3) the SD ratio between the membrane potential in the prestimulus phase and in the 500-ms time window following the whisker stimulation onset was >2 and the Pearson correlation coefficients between half subsets of recordings > 0.5 in at least 1 stimulus direction; 4) the difference in the mean membrane potential during the prestimulus phase between the no-light and the light condition was <10 mV for each stimulus direction; 5) the difference between the mean membrane potential in the prestimulus phase during the duration of the whole experiment was <20 mV. Cells meeting the requirements described above but with <10 trials for each condition were not considered.

### Experimental Design and Statistical Analysis

Statistical analysis was performed using OriginPro 9.1 (OriginLab Corporation), GraphPad Prism 7.03 (GraphPad Software) and Python. Data were tested for normality with both D’Agostino and Pearson, and Shapiro–Wilk normality tests. In case of normally distributed populations, the 2-tailed paired Student’s *t*-test or the 1-way analysis of variance (ANOVA) repeated measures with Bonferroni post hoc test were used to confront, respectively, 2 or more datasets. For not normally distributed populations, the 2-tailed Wilcoxon signed rank test or the Friedman test with Dunn’s post hoc test were used to compare, respectively, 2 or more datasets. All tests were 2-sided, unless otherwise stated. Values are shown as mean ± standard error of mean (SEM) with ^*^indicating *P* ≤ 0.05, ^*^^*^indicating *P* ≤ 0.01, ^*^^*^^*^indicating *P* ≤ 0.001, unless otherwise stated. No statistical methods were used to predetermine sample size, but we collected sample sizes of cells and animals similar to those reported in previous publications (Brecht et al. 2003; Pluta et al. 2015). The numbers of cells and animals for Figures 2–6 and Supplementary Figures 2–6 and the number of imaged section for Figure 1 and Supplementary Figure 1 are provided in the corresponding figures and figure legends. All collection and analysis were not blind to experimental conditions. Criteria for data inclusion are described in the above sections.

### Code Accessibility

Custom codes used in the current study are available from the Lead Contact upon request.

## Results

### Selective Expression of Halorhodopsin in L4 Excitatory Neurons of the Mouse Barrel Cortex

To investigate whether L2/3 cells inherit direction selectivity from L4, we first targeted the expression of the inhibitory opsin eNpHR3.0-eYFP (Halo-eYFP) (Gradinaru et al. 2010) to L4. To target excitatory neurons in L4, we used the Scnn-Cre mouse line, which expresses Cre recombinase specifically in L4 glutamatergic neurons of primary sensory cortices (Madisen et al. 2010). To easily identify Scnn-Cre^+^ mice and Scnn-Cre^+^ cells, this line was bred with a Cre-dependent reporter strain conditionally expressing the red fluorescent protein TdTom. Bigenic newborn transgenic animals (Scnn × TdTom) were injected in S1 with a Cre-dependent AAV encoding Halo-eYFP (Gradinaru et al. 2010) (see Materials and Methods and Fig. 1*A*). After 4–5 weeks of postinjection, expression of the fluorescent reporter (eYFP) was clearly visible in neurons located in L4 of S1bf (Fig. 1*B*,*C*, right). To quantify the AAV-mediated transgene expression efficacy in S1bf, we acquired confocal images of fixed cortical sections from Scnn × TdTom mice injected with an AAV carrying a flex construct for cytosolic eGFP (Oh et al. 2014). We found that approximately 80% of the cells positive for the neuronal marker, NeuN, within L4 barrels also expressed TdTom and that approximately 95% of TdTom^+^ cells were eGFP^+^, Fig. 1*D*–*F*, see Materials and Methods for details). Negligible number of eGFP^+^ cells was observed in L2/3 (Supplementary Fig. 1). Intracellular patch-clamp recordings in coronal slices demonstrated that all the L4 TdTom^+^ cells showed a regular firing pattern typical of excitatory neurons (data not shown). These data together with previous findings (Pluta et al. 2015) demonstrate that this animal model can be used to specifically manipulate the activity of the large majority of L4 excitatory neurons in S1bf.

### Optogenetic Inhibition of Whisker-Evoked Response in L4 Excitatory Neurons

To test the functionality of the inhibitory opsin Halo*,* we first performed blind patch-clamp recordings from L4 Halo-expressing neurons combined with yellow light illumination (λ = 594 nm) during whisker deflection in urethane-anesthetized mice (Fig. 2*A*). In whisker-stimulation experiments, all but one whiskers contralateral to the injected hemisphere were trimmed and we performed passive stimulation of the spared whisker by means of a piezoelectric actuator (see Materials and Methods for details). The stimulation consisted of a step-and-hold deflection (on phase) (Brecht and Sakmann 2002b), lasting 500 ms. At the end of the deflection, the whisker was brought back to its original position (off phase, Fig. 2*A*). Patch-clamp recordings were performed from L4 excitatory neurons in the cortical barrel corresponding to the stimulated whisker, which was previously identified using IOI (see Materials and Methods for details). We found that whisker deflection alone evoked membrane depolarization of L4 excitatory cells (Fig. 2*B*, top). Yellow light illumination (duration: 1 s) during whisker deflection significantly hyperpolarized the membrane potential of recorded neurons compared with the whisker stimulation alone (Fig. 2*B*, bottom and Fig. 2*C*,*D*).

In addition, we performed 2-photon targeted juxtasomal recordings from L4 TdTom^+^ cells in anesthetized mice injected with Halo and we quantified the effect of the inhibitory opsin in modulating L4 whisker-evoked spiking activity (Fig. 2*E*). We found that yellow light illumination significantly decreased whisker-evoked spiking activity (Fig. 2*F*,*G*). We also quantified the effect of optogenetic inhibition of L4 on the spontaneous spiking. Yellow light illumination significantly reduced baseline spike frequency (Fig. 2*H*–*J*). Halo activation thus effectively inhibits sensory-evoked and spontaneous spiking activity of L4 neurons.

### Optogenetic Inhibition of L4 Does Not Abolish L2/3 Response to Whisker Deflection

To measure the contribution of the L4 to the subthreshold response of L2/3 to whisker stimulation, we recorded the membrane potential of L2/3 neurons during whisker deflection and during combined sensory stimulation and optogenetic inhibition of L4 principal cells initially in anesthetized mice (duration of optogenetic inhibition: 1 s; Fig. 3*A*). Whisker-evoked responses were divided in 3 phases: early, late, and off (Brecht and Sakmann 2002b; Sachidhanandam et al. 2013). Optogenetic inhibition of L4 significantly decreased the whisker-evoked response of L2/3 pyramidal cells during the late (from 100 to 500 ms after whisker deflection onset) and off (from 10 to 400 ms after the end of whisker stimulation) phases of the whisker response (Fig. 3*B*,*C* and Fig. 3*D* center and right). Surprisingly, the early phase of the response (from 10 to 100 ms after whisker deflection onset) was not significantly affected by optogenetic inhibition of L4 (Fig. 3*B*,*C* and Fig. 3*D* left). To control that the decreased sensory response was due to optogenetic inhibition of L4 neurons and to exclude an effect of light per se, we repeated the same experiments described above in mice that did not express Halo (Supplementary Fig. 2*A*). Whisker stimulation alone depolarized the membrane potential of supragranular pyramidal cells (Supplementary Fig. 2*B* top, *C*), but yellow light illumination had no effect on the sensory-evoked response of L2/3 neurons (Supplementary Fig. 2*B* bottom, *C*,*D*). Taken together, these findings demonstrate that optogenetic inhibition of L4 differentially affects the various phases of the subthreshold whisker-evoked response in L2/3 principal cells, with late and off responses being decreased and early responses being surprisingly unaffected. This latter observation implies that L2/3 cells are only partially driven by L4 during sensory stimulation in the mouse S1bf.

### Optogenetic Inhibition of L4 Does Not Alter Spontaneous Cortical Activity

S1 circuits are active even in the absence of sensory inputs (Petersen et al. 2003; Luczak et al. 2007; Beltramo et al. 2013). A major component of this spontaneous activity is the up and down state-like transitions, which characterize cortical neurons under many forms of anesthesia or during quiet wakefulness (Petersen et al. 2003; Crochet and Petersen 2006; Crunelli and Hughes 2010). Up states are triggered by sensory stimuli and direct electrical stimulation of the thalamus (Petersen et al. 2003; MacLean et al. 2005; Civillico and Contreras 2012; Reig et al. 2015), suggesting that these stimuli may induce up state transitions in L2/3 neuron via L4. However, several studies pointed to deep infragranular layers as fundamental in the generation and propagation of up states (Sanchez-Vives and McCormick 2000; Chauvette et al. 2010; Wester and Contreras 2012; Beltramo et al. 2013). To test whether L4 was involved in the modulation of up and down state-like activity (Petersen et al. 2003; Crochet and Petersen 2006; Crunelli and Hughes 2010), we optogenetically inhibited L4 principal cells (optogenetic stimulus duration: 1 s) while performing patch-clamp recordings from L2/3 principal cells in anesthetized mice. We first considered only recordings in which the membrane potential of cortical neurons displayed clear up and down state rhythmic transitions (see Materials and Methods for definitions), automatically detected using a custom algorithm (Zucca et al. 2017; Zucca et al. 2019). We observed no significant effect of the optogenetic inhibition of L4 on the up and down state transitions of L2/3 neurons in S1bf. The average membrane potential of cortical neurons in the supragranular layer did not significantly change upon yellow light illumination, either when light occurred during an ongoing down state (Fig. 4*A* and Fig. 4*C* left) or during an ongoing up state (Fig. 4*B* and Fig. 4*D* left) (see Materials and Methods for analysis details). Moreover, the duration of the ongoing down or up state (Fig. 4*E*,*F*, left) as well as the total time spent by the recorded neurons in either down or up states (Fig. 4*G*–*J*, left) were unaffected by optogenetic inhibition of L4. We extended these experiments in awake head-restrained animals during periods of quiet wakefulness (Petersen et al. 2003; Crochet and Petersen 2006). Similar to the results in anesthetized animals, no significant difference in the spontaneous bimodal state dynamics of L2/3 principal cells was observed during L4 optogenetic inhibition compared with the controls in all the considered parameters (Fig. 4*C*–*J*, right). Since the definition of up and down states-like dynamics can be nontrivial in awake mice, we also classified membrane potential dynamics based on the ratio between low- and high-frequency components of the membrane potential dynamics (see Material and Methods for definition). Periods with high value (>50) of the ratio were classified as synchronized periods and time windows with lower ratio (<30) were classified as desynchronized periods. We found no effects of L4 optogenetic inhibition on the cell’s average membrane potential and its variance in both synchronized and desynchronized time periods (Supplementary Fig. 3). These results suggest that modulation of cortical subthreshold spontaneous dynamics in L2/3 is largely dependent on circuits other than L4.

### Widespread Cortical Silencing Through the Optogenetic Activation of Parvalbumin-positive (PV) Interneurons Suppresses Whisker-Evoked Response in L2/3

L2/3 whisker responses that were not suppressed by L4 optogenetic inhibition may be due to direct innervation by excitatory inputs from cortical layers other than L4 or to longer range excitatory inputs (e.g., thalamo-cortical inputs). To start discriminating among these possibilities, we performed in vivo patch-clamp recordings from L2/3 principal cells during whisker stimulation while optogenetically silencing cortical activity by optogenetic activation of PV interneurons expressing Channelrhodopsin 2 (ChR2; Nagel et al. 2003; Boyden et al. 2005; Supplementary Fig. 4*A*). Optogenetic activation of PV cells has been shown to provide widespread cortical inhibition (Lien and Scanziani 2013). ChR2 was expressed selectively in PV cells by injecting AAVs carrying a double-floxed ChR2 construct into PV-Cre × TdTom mice. We found that optogenetic activation of PV cells (stimulus duration: 1 s) almost completely abolished whisker responses in L2/3 principal neurons (Supplementary Fig. 4*B*,*C*). In all the 3 temporal windows considered (early, late, and off phases), the membrane potential depolarization elicited by whisker stimulation was largely reduced (Supplementary Fig. 4*C*,*D*).

### L4 Modulates Subthreshold Direction Tuning of L2/3 Pyramidal Cells

The experiments described in Figure 3 and Supplementary Figure 4 demonstrate that L2/3 subthreshold response to whisker deflection contains a L4-dependent component but also a L4-independent component. Since L4 cells display tuning of their response depending on the direction of the whisker deflection (Simons and Carvell 1989; Brecht and Sakmann 2002a; Bruno and Simons 2002; Lee 2004; Ramirez et al. 2014), this observation suggests the possibility that the L4-sensitive component influences the direction tuning of L2/3. To test this possibility, we performed whole-cell current-clamp recordings from L2/3 pyramidal neurons while deflecting the principal whisker in 4 different angular directions under control conditions and during optogenetic inhibition of L4 initially in anesthetized animals (see Materials and Methods for experimental details). Under control conditions, the amplitude of the postsynaptic potential during whisker stimulation varied as a function of the angle of whisker deflection (Fig. 5*A*; Brecht et al. 2003). We ranked from 1 to 4 the directions of the whisker deflection on the bases of the amplitude of the corresponding evoked response in the early phase under control conditions (Whisker; Table 1). We defined as 1 (preferred) the direction that evoked the highest whisker-evoked response in the early phase and as 4 (nonpreferred) the direction that evoked the lowest sensory response. Intermediate directions were ranked as 2 and 3 for the second highest and the third highest response, respectively (Fig. 5*A*). During whisker stimulation, we observed a significant difference in the amplitude of the subthreshold whisker response depending on the ranks (Table 1, top left). When whisker stimulation was paired with optogenetic inhibition of L4 and the stimulus was presented in the preferred direction (rank 1), we found that L4 optogenetic inhibition significantly decreased the whisker-evoked response in the early phase (Fig. 5*B*,*C*, top) as well as in the late and off response phases (Supplementary Fig. 5*A*,*B*). Optogenetic inhibition of L4 in the rank 2 and 3 conditions had no significant effect on any phase of the L2/3 response to the whisker stimulus (Fig. 5*B*,*C*, middle panels and Supplementary Fig. 5*C*–*F*). Interestingly, in the least preferred direction (rank 4) optogenetic inhibition of L4 increased the early (Fig. 5*B*,*C*, bottom) and late phase responses with no effect in the off phase (Supplementary Fig. 5*G*,*H*). As a consequence, no significant difference in the amplitude of the whisker-evoked response across ranks was found when whisker stimulation was combined with optogenetic inhibition of L4 (Table 1, top right). To confirm that L4 optogenetic inhibition had an effect on the direction tuning of L2/3 cells, we computed the direction selectivity index (DSI) for L2/3 responses in the early phase (see Materials and Methods for details). We found that optogenetic inhibition of L4 significantly decreased the DSI, indicating L4 modulates the direction selectivity tuning of L2/3 pyramidal cells (Fig. 5*D*).

**Table 1:** Direction-dependency of subthreshold whisker-evoked response of L2/3 principal cells

**Anesthetized**					
Whisker			Whisker + Light		
Whisker response (mV)		Post hoc test	Whisker response (mV)		Post hoc test
Rank 1	7.43 ± 1.11	Rank 1 vs. Rank 2: ^*^^*^^*^ Rank 1 vs. Rank 3: ^*^^*^^*^ Rank 1 vs. Rank 4: ^*^^*^^*^ Rank 2 vs. Rank 3: ^*^^*^ Rank 2 vs. Rank 4: ^*^^*^^*^ Rank 3 vs. Rank 4: ^*^^*^^*^	Rank 1	6.20 ± 1.15	Rank 1 vs. Rank 2: NS Rank 1 vs. Rank 3: NS Rank 1 vs. Rank 4: NS Rank 2 vs. Rank 3: NS Rank 2 vs. Rank 4: NS Rank 3 vs. Rank 4: NS
Rank 2	6.40 ± 1.05		Rank 2	5.59 ± 0.97	
Rank 3	5.16 ± 0.89		Rank 3	5.78 ± 1.03	
Rank 4	4.08 ± 0.79		Rank 4	6.12 ± 1.14	
**Awake**					
Whisker			Whisker + Light		
Whisker response (mV)		Post hoc test	Whisker response (mV)		Post hoc test
Rank 1	5.83 ± 0.64	Rank 1 vs. Rank 2: ^*^ Rank 1 vs. Rank 3: ^*^^*^^*^ Rank 1 vs. Rank 4: ^*^^*^^*^ Rank 2 vs. Rank 3: ^*^ Rank 2 vs. Rank 4: ^*^^*^^*^ Rank 3 vs. Rank 4: ^*^	Rank 1	4.74 ± 0.64	Rank 1 vs. Rank 2: NS Rank 1 vs. Rank 3: NS Rank 1 vs. Rank 4: ^*^^*^ Rank 2 vs. Rank 3: NS Rank 2 vs. Rank 4: NS Rank 3 vs. Rank 4: NS
Rank 2	4.77 ± 0.61		Rank 2	4.14 ± 0.63	
Rank 3	4.02 ± 0.58		Rank 3	4.36 ± 0.63	
Rank 4	3.15 ± 0.57		Rank 4	3.66 ± 0.59	

Notes: Top: average ± SEM of whisker-evoked responses (Whisker response) in the early phase of rank 1 to rank 4 whisker-deflection directions during whisker stimulation (Whisker) and whisker stimulation combined with optogenetic inhibition of L4 (Whisker + Light) in anesthetized animals. *n* = 21 cells from 12 animals. One-way ANOVA repeated measures with Bonferroni post hoc test, *P* = 3E-8 for Whisker. Friedman test with Dunn’s post hoc test, *P* = 0.99 for Whisker + Light. Bottom: same as above for awake head-restrained animals. *n* = 24 cells from 11 animals. Friedman test with Dunn’s post hoc test, *P* = 2E-15 for Whisker. Friedman test with Dunn’s post hoc test, *P* = 6E-3 for Whisker + Light. NS, not significant.

We finally asked if the direction-dependent effect of optogenetic inhibition of L4 on the subthreshold whisker response observed under anesthesia was also observed in nonanesthetized conditions. To this aim, we performed patch-clamp recordings from L2/3 principal neurons in awake head-fixed animals, which were habituated to quietly sit on the experimental setup (see Materials and Methods, Fig. 6; Supplementary Fig. 6; Table 1, bottom). Similarly to what was observed in anesthetized animals, we found a significant difference in the whisker-evoked response across ranks (Table 1, bottom left). This difference was reduced by optogenetic inhibition of L4 (Table 1, bottom right). Moreover, optogenetic inhibition of L4 significantly reduced whisker-evoked response of excitatory supragranular neurons in the preferred direction (rank 1) in the early, late, and off phases (Fig. 6*B*,*C*, top and Supplementary Fig. 6*A*,*B*). We also observed that the L2/3 sensory response decreased during optogenetic inhibition of L4 neurons in the early, late, and off phases also for the rank 2 direction and in the late phase for the rank 3 direction (Fig. 6*B*,*C*, middle panels and Supplementary Fig. 6*C*–*F*). Optogenetic inhibition of L4 increased the supragranular neurons sensory response in the early phase (Fig. 6*B*,*C*, bottom) for the least preferred (rank 4) direction, but not in the late and off response phases (Supplementary Fig. 6*G*,*H*). The DSI of L2/3 pyramidal cells was reduced by optogenetic inhibition of L4 (Fig. 6*D*). Taken together, the results of these experiments in both anesthetized and nonanesthetized animals demonstrate that L4 performs stimulus feature-specific control of L2/3 response by modulating direction selectivity tuning.

## Discussion

We combined optogenetic inhibition of L4 S1bf excitatory neurons with intracellular recording in anesthetized and awake head-restrained mice during whisker stimulation to elucidate the origin of the subthreshold cortical representation of the principal whisker stimulation in superficial L2/3 excitatory cells*.* In L2/3, subthreshold responses to whisker inputs are much more robust and reliable compared with suprathreshold responses (Brecht et al. 2003; Crochet and Petersen 2006; de Kock et al. 2007; O'Connor, Peron, et al. 2010b; Crochet et al. 2011; Barth and Poulet 2012; Vecchia et al. 2020), enabling the study of the integration of different inputs (Petersen and Crochet 2013). We found that L2/3 responses were reduced as a consequence of optogenetic inhibition of L4. However, a large subthreshold component of the L2/3 response surprisingly survived optogenetic inhibition of L4, including the response within the first 100 ms from whisker stimulation (early phase; Fig. 3). Since L4 receives direct angular-tuned inputs from the VPM and shows strong directional tuning (Brecht and Sakmann 2002a; Bruno and Simons 2002; Bruno et al. 2003; Lee 2004), we further investigated the effect of optogenetic inhibition of L4 to the subthreshold direction tuning of the L2/3 response.

In anesthetized mice, L2/3 displayed subthreshold direction selectivity, similar to that reported previously for anesthetized and lightly sedated rats (Fig. 5 and Table 1, top; Brecht et al. 2003; Ramirez et al. 2014). The reduction in L2/3 response upon optogenetic inhibition of L4 was dependent on the direction of the whisker deflection. When optogenetic inhibition of L4 was performed during the stimulation along the preferred whisker direction, the amplitude of the subthreshold neuronal response in all the phases was reduced. In contrast, when optogenetic inhibition of L4 was performed during whisker stimulation along the least preferred whisker direction, we observed a significant increase in the early (first 100 ms) and late (100–500 ms) phases of the L2/3 response (Fig. 5 and Supplementary Fig. 5). These data suggest that L4 activity has an antithetical direction-dependent effect on the early response phase of L2/3 cells. Consequently, the direction tuning of the neurons in L2/3 quantified as DSI significantly decreased upon optogenetic inhibition of L4 (Fig. 5*D*). These observations are compatible with the lack of effect on the early phase of L2/3 response during optogenetic inhibition of L4 when the directional component of the stimulus was not considered (Fig. 3), because the opposing direction-dependent effects are averaged out when all directions are pulled together.

Our conclusions on the effect of optogenetic inhibition of L4 on L2/3 subthreshold activity rely on the effective silencing of L4 during light presentation. We made important control experiments to verify this. First, we found that the inhibitory opsin Halo was specifically expressed in the vast majority of L4 excitatory neurons (Fig. 1) with negligible leakage in L2/3 (Supplementary Fig. 1), similarly to what observed in a previous study using an analogous experimental approach to silence L4 (Pluta et al. 2015). Second, we controlled for opsin functionality and we observed efficient suppression of whisker-evoked firing in L4 Halo-expressing cells upon illumination (Fig. 2). Third, to exclude the possibility that the response of L2/3 excitatory cells located above one barrel could be indirectly influenced by L4 activity in a neighboring barrels through horizontal L2/3–L2/3 connections (Bureau et al. 2006; Adesnik and Scanziani 2010), we used a large fiber optic (fiber diameter, 0.94 mm) positioned approximately 1 mm above the pia. This resulted in an illuminated area of approximately 2 mm^2^.

Since the direction-dependent properties of the subthreshold response of L2/3 to whisker stimulation are poorly described in nonanesthetized mice, we extended the previous experiments in awake animals. We found that L2/3 subthreshold response displayed direction dependence (Fig. 6*D* and Table 1, bottom). Moreover, optogenetic inhibition of L4 resulted in a direction-dependent effect on the subthreshold L2/3 response. We observed an overall decrease of the response in the preferred direction and an increase in the early phase of the least preferred direction (Fig. 6). Compared with experiments in anesthetized animals, in awake mice we found a decreased response also in all the phases for the second highest preferred direction and in the late phase for the third highest preferred direction (Fig. 6 and Supplementary Fig. 6). This resulted in the reduction of the DSI of L2/3 principal cells upon optogenetic inhibition of L4 (Fig. 6*D*).

The L4-independent component of the subthreshold L2/3 response may be due to short-range connections in S1bf, for example, intercolumnar L2/3–L2/3 connections or intracolumnar infragranular–supragranular connections (Feldmeyer 2012) or by direct long-range inputs originating outside S1bf from, for example, thalamic fibers (Petreanu et al. 2009; Meyer et al. 2010; Oberlaender et al. 2012; Audette et al. 2018), secondary somatosensory cortex (Aronoff et al. 2010), primary motor cortex (Petreanu et al. 2009; Aronoff et al. 2010; Mao et al. 2011), visual and auditory primary cortices (Sieben et al. 2013; Stehberg et al. 2014; Henschke et al. 2015), and contralateral S1 (Petreanu et al. 2007). The observation that optogenetic activation of cortical PV interneurons (Lien and Scanziani 2013) almost completely suppressed L2/3 responses (Supplementary Fig. 4) may suggest that the response elicited in L2/3 by the whisker input relied mostly on the short- rather than long-range inputs. This conclusion is based on the assumption that the shunting effect and the reduced input resistance induced in principal L2/3 cells by PV optogenetic activation do not completely prevent the membrane depolarization induced by the activation of long-range fibers. Although the aforementioned assumption was considered realistic in previous studies investigating long-range inputs to cortical cells (Li, Li, et al. 2013b; Li, Ibrahim, et al. 2013a; Lien and Scanziani 2013; Cohen-Kashi Malina et al. 2016), we cannot completely rule out a role of long range fibers (e.g., direct thalamic inputs in L3) in driving direction-independent subthreshold responses in L2/3. Within this framework, it is interesting to note that a role of direct thalamocortical input in shaping L2/3 response to the sensory input has been proposed based on patch-clamp recordings in the binocular region of the rat visual cortex in L4 and L2/3 (Medini 2011). In addition, previous work in S1bf showed that L2/3 neurons integrate both direct lemniscal (Sermet et al. 2019) and paralemniscal thalamic inputs (Jouhanneau et al. 2014; Zhang and Bruno 2019) as well as L4 inputs (Petersen and Sakmann 2001; Lefort et al. 2009). Moreover, both sub- and suprathreshold sensory-evoked responses of L2/3 pyramidal cells are modulated by horizontally L2/3–L2/3 projections across barrel-columns (Adesnik and Scanziani 2010) as well as by cross-modal inputs (Iurilli et al. 2012; Sieben et al. 2013; Zhang et al. 2020). In L2/3 of S1bf, the excitatory feedback from the secondary somatosensory cortex (Kwon et al. 2016), potentially modulated by a cortico-thalamo-cortical circuit (L5B-Pom-S2, Theyel et al. 2010), may also significantly contribute to subthreshold response.

Another potential source of the L4-independent response of L2/3 may be deep infragranular cortical laminae, especially L5. L5 excitatory neurons directly synapse onto L2/3 (Shepherd et al. 2005; Bureau et al. 2006; Lefort et al. 2009; Oberlaender et al. 2011; Staiger et al. 2015). Excitatory cells in infragranular layers receive direct VPM inputs and are activated by sensory stimuli with latencies similar compared with that of L4 cells (Constantinople and Bruno 2013). Moreover, L5 sharpens the sensory responses within the barrel cortical column including L2/3 (Vecchia et al. 2020). A potential role of L5 in contributing to the subthreshold L2/3 response would also be compatible with the observation that optogenetic inhibition of L4 causes an increase in the amplitude of the least preferred direction. In fact, previous work demonstrated that L4 suppresses the excitability of L5 through the activation of specific inhibitory circuits (Pluta et al. 2015) and that L5 forms direct excitatory synapses on L2/3 (Lefort et al. 2009; Vecchia et al. 2020). Optogenetic inhibition of L4 could thus increase firing in L5, leading to increased L5 excitatory input to L2/3. Future experiments employing transgenic mouse lines that selectively target the majority of the excitatory cells in infragranular layers will be needed to test these hypotheses.

It is important to note that, although statistically significant, the effects we observed on the membrane potential of L2/3 cells following optogenetic inhibition of L4 had small amplitude. This observation raises the important question of whether the phenomenon we described in this study may have functional consequences at higher levels (e.g., in a behavioral task). Although we cannot provide a conclusive answer based on the data provided in this study and future experiments will be required to address this question, it should be noted that the direction tuning of principal neurons is considered one important feature of S1bf circuits (Bale and Maravall 2018). Although it is not clear if orientation tuning of S1bf neurons is used by the animal to drive behavior (Adibi 2019), rats learn to discriminate about the deflection of one whisker in different orientation (Schriver et al. 2018) and mice discriminate object angle at the behavioral level (Kim et al. 2020). It is interesting to note that in this latter study >60% of neurons tuned for the object angle in the primary somatosensory cortex during the task were found to be tuned for the direction of passive whisker deflection (Kim et al. 2020). Moreover, neurons tuned for both the direction of passive whisker deflection and for the object angle displayed a weak relationship between these 2 tuning parameters (Kim et al. 2020). Although tuning to the passive deflection direction was neither necessary nor sufficient to for object-angle tuning during active whisker touch, those results demonstrate partial overlap between neurons tuned to the direction of the passive whisker deflection and neurons tuned to the object angle in a behavioral task. Our results indicate that optogenetic inhibition of L4 has small effect on the membrane potential dynamics of L2/3 neurons, but these small-amplitude changes result in the significant modification the tuning properties of L2/3 cells for the direction of whisker deflection. Our study thus contributes to dissect out the mechanisms underlying direction selectivity coding in S1bf circuits. Given the partial overlap between the tuning properties for direction and object angle selectivity, direction tuning encoding in L2/3 may contribute to certain types of whisker-dependent behaviors.

S1 circuits are active even in the absence of sensory inputs (Petersen et al. 2003; Luczak et al. 2007; Beltramo et al. 2013; Zucca et al. 2019). Major components of these spontaneous activities are oscillations in the frequency band 0.5–4 Hz, which represents the dominant cortical rhythm observed during quite wakefulness, deep stages of NREM sleep, and under several types of anesthesia (Steriade et al. 1993a, 1993b; Petersen et al. 2003; Crochet and Petersen 2006; Luczak et al. 2007; Vyazovskiy et al. 2009; Crunelli and Hughes 2010; Vyazovskiy et al. 2011; Gonzalez-Rueda et al. 2018). From the intracellular point of view some of these spontaneous activities are characterized by bistable membrane potential fluctuations, the so-called up and down states. Up and down states are observed in many subcortical regions, including the thalamus. Thalamic inputs are known to regulate spontaneous cortical network activity (Poulet et al. 2012; Lemieux et al. 2014; Lemieux et al. 2015; Zucca et al. 2019) and sensory stimulation or direct electrical stimulation of the thalamus trigger cortical up state generation (Petersen et al. 2003; MacLean et al. 2005; Civillico and Contreras 2012; Reig et al. 2015). However, we found no effect of optogenetic inhibition of L4 on subthreshold spontaneous membrane dynamics of L2/3 in both anesthetized animals and awake animals (Fig. 4 and Supplementary Fig. 3). These results suggest that thalamic control of spontaneous cortical dynamics may be largely independent on the activation of L4 and it could instead be achieved through activation of infragranular layers. In agreement with this hypothesis, deeper laminae have been reported to be crucial for the generating and propagating slow spontaneous cortical dynamics (Silva et al. 1991; Sanchez-Vives and McCormick 2000; Sakata and Harris 2009; Chauvette et al. 2010; Wester and Contreras 2012; Beltramo et al. 2013).

The quest to understand how sensory perception arises from the coordinated action of brain circuits requires the detailed dissection of how different features of the sensory stimulus are encoded in specific neuronal populations and how this information flows from pre- to postsynaptic networks. Our results support the idea that L4 principal cells modulate how specific features of the sensory stimulus (e.g., the direction of the whisker deflection) are encoded in L2/3, but these findings also show that response of L2/3 excitatory cells is largely influenced by L4-independent depolarizing inputs. These findings call for a reconsideration of the canonical cellular model of cortical circuits and contribute to shed light on the network mechanisms underlying a fundamental property of the cortex that is how sensory information is transferred and processed by cortical circuits.

## Funding

European Research Council (ERC, NEURO-PATTERNS); National Institutes of Health: Brain Initiative (U01 NS090576, U19 NS107464).

## Notes

We thank Y. Zerlaut for preliminary analysis, K. Deisseroth for pAAV-Ef1a-DIO eNpHR 3.0-EYFP (Addgene plasmid #26966) and pAAV-EF1a-double floxed-hChR2(H134R)-mCherry-WPRE-HGHpA (Addgene plasmid #20297), and H. Zeng for AAV-pCAG-FLEX-EGFP-WPRE (Addgene plasmid #51502). *Conflict of Interest*: None declared.

## Supplementary Material

Varani_et_al_Supplementary_Materials_bhab297
