# Supplementary material for: Stimulus Feature-Specific Control of Layer 2/3 Subthreshold Whisker Responses by Layer 4 in the Mouse Primary Somatosensory Cortex
Source: Cereb Cortex. 2021 Aug 27;32(7):1419–36. doi: 10.1093/cercor/bhab297 (PMC8971086; doi:10.1093/cercor/bhab297)
Supplement: Varani_et_al_Supplementary_Materials_bhab297 [file varani_et_al_supplementary_materials_bhab297.pdf]

Stimulus feature-specific control of layer 2/3 subthreshold whisker responses by layer 4 in the mouse primary somatosensory cortex

Supplementary Materials

Containing six figures with legends

Stefano Varani, Dania Vecchia, Stefano Zucca, Angelo Forli, Tommaso Fellin*

Optical Approaches to Brain Function Laboratory, Istituto Italiano di Tecnologia, 16163 Genova, Italy.

* corresponding author:

Tommaso Fellin, Optical Approaches to Brain Function Laboratory, Istituto Italiano di Tecnologia, Via Morego 30, 16163 Genova, Italy, tel: +39 010 71781549, fax:+39 010 71781230, email: tommaso.fellin@iit.it

Running title: Feature-dependent control of L2/3 by L4

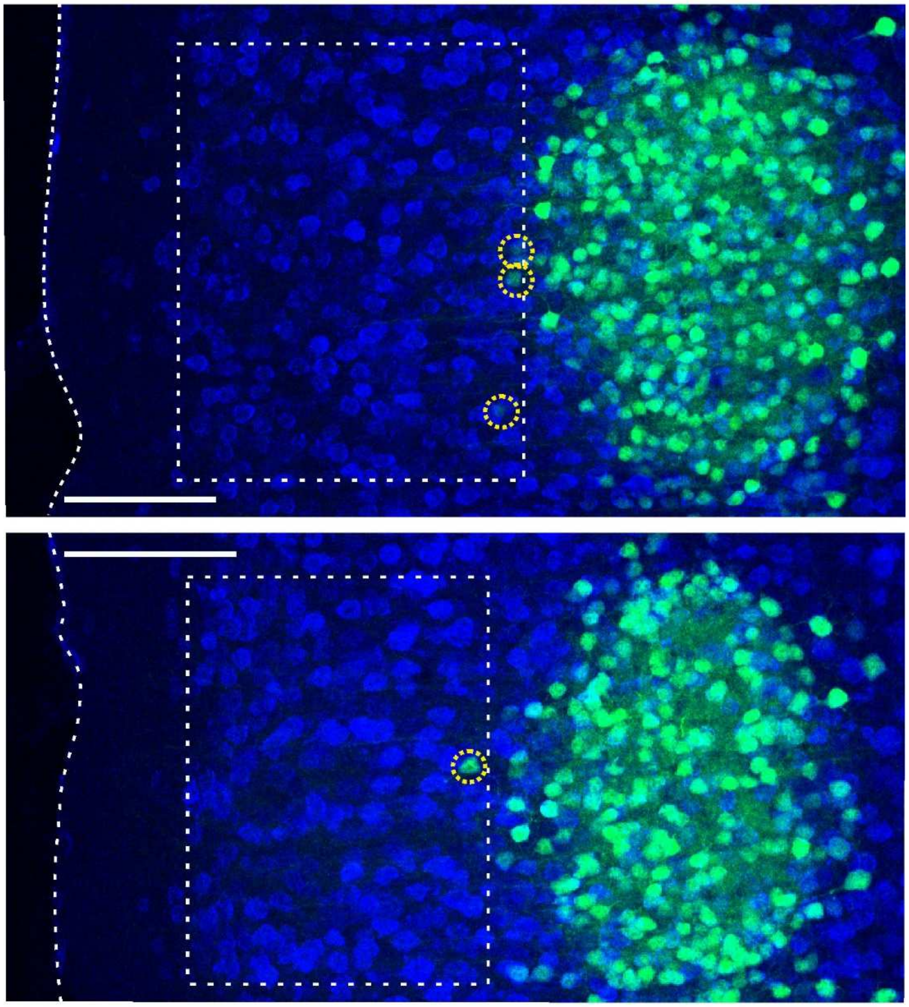

Supplementary Figure 1. Expression of Cre-recombinase in L2/3 of S1bf in Scnn-Cre mice. Confocal images of two coronal sections showing the maximal intensity projection of a z-stack showing NeuN staining (blue) and eGFP staining (green) in the S1bf of Scnn-Cre mice injected with AAVs carrying the floxed eGFP construct. White dashed lines indicate the cortical surface. White dashed rectangles identify the boundaries of L2/3 which were used for cell count in the indicated samples. The yellow dotted circles indicate eGFP$^+$ cells located in L2/3. Scale bars: 100 µm. The average density of eGFP$^+$ cells was 0.8 cells per 100 µm x 100 µm x 100 µm of L2/3 volume. The majority (~ 70 %) of the few cells which were counted as L2/3 were located at the border between L2/3 and L4. The quantification was done averaging data across 44 barrel related columns in 23 sections from 2 animals.

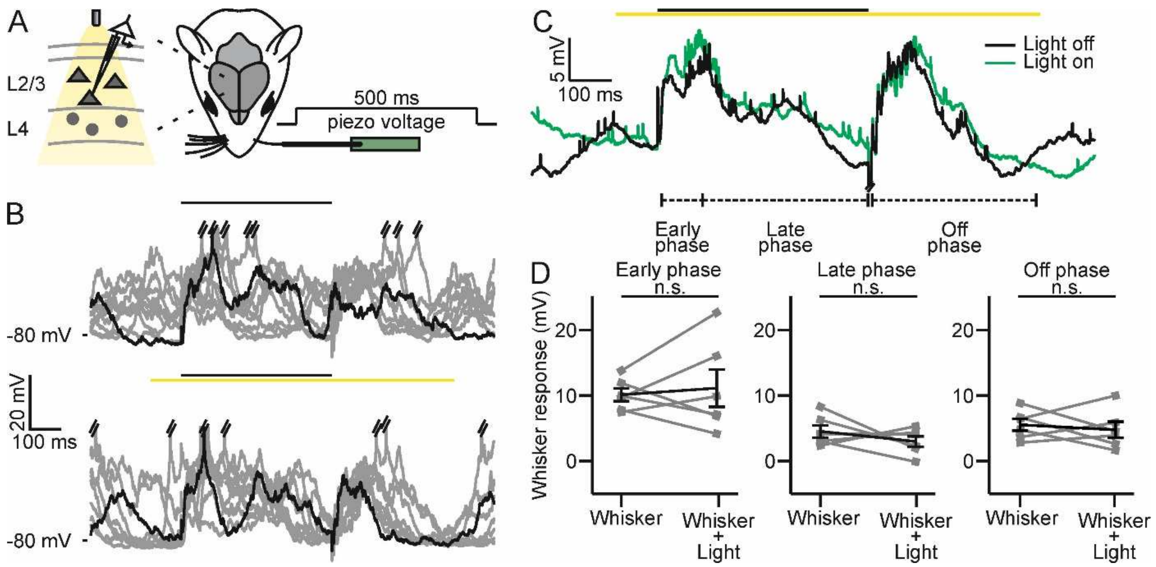

Supplementary Figure 2. No effect of optogenetic manipulation on L2/3 responses to whisker deflection in mice that do not express Halo. (A) Schematic representation of the experimental setup for intracellular recordings in L2/3 pyramidal neurons in anesthetized mice. No Halo expression in L4. Sensory and optogenetic stimulation were performed as described in Figure 2A. (B) Ten representative traces showing the membrane potential response of a L2/3 cell during whisker stimulation in the absence (top) and presence (bottom) of yellow light illumination. (C) Average membrane potential response of 30 trials in the absence (black) and presence (green) of light illumination for the cell shown in B. Early, late, and off temporal windows are indicated below the traces (dashed lines). (D) Membrane potential values of the whisker-evoked depolarization in L2/3 pyramidal neurons during whisker stimulation in the absence (Whisker) and presence of light illumination (Whisker + Light) for the early (left), late (center), and off phase (right) temporal windows. n = 6 cells from 4 animals. Wilcoxon signed rank test: p = 0.84 for early phase; p = 0.31 for late phase; p = 0.56 for off phase.

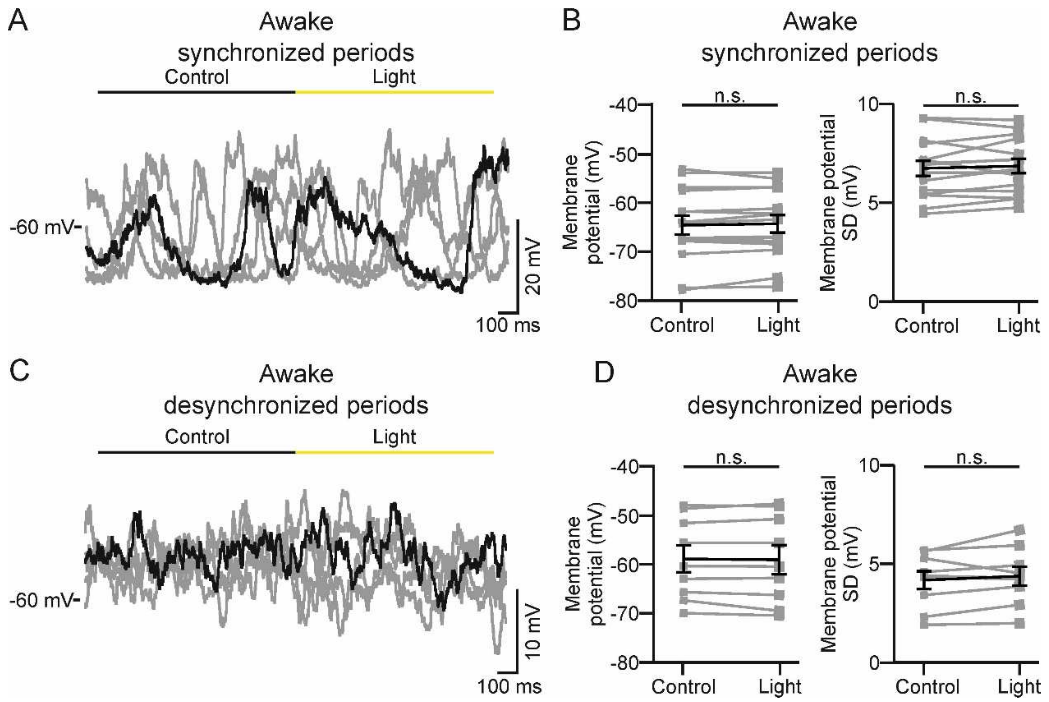

Supplementary Figure 3. Optogenetic inhibition of L4 does not affect spontaneous membrane potential dynamics in L2/3 pyramidal cells recorded during synchronized and desynchronized waking periods. (A) Representative current-clamp recordings showing the membrane potential of a L2/3 pyramidal cell during spontaneous synchronized activity in an awake head-fixed animal. Yellow light stimulation is indicated by the yellow bar. Five trials are shown (one in black and four in grey). (B) Left: average membrane potential for synchronized periods during the control time window (Control) and during optogenetic inhibition of L4 (Light) in awake head-fixed animals. Paired Student's *t*-test: p = 0.30, n = 15 cells in 7 animals. Right: same as in the left panel but for the average standard deviation (SD) of the membrane potential. Paired Student's *t*-test: p = 0.40, n = 15 cells in 7 animals. (C) Same as in A, but for traces during spontaneous desynchronized activity in an awake animal. (D) Same as in B for desynchronized periods. Paired Student's *t*-test: p = 0.60 and p = 0.27 for the left and right panels, respectively. n = 9 cells in 6 animals.

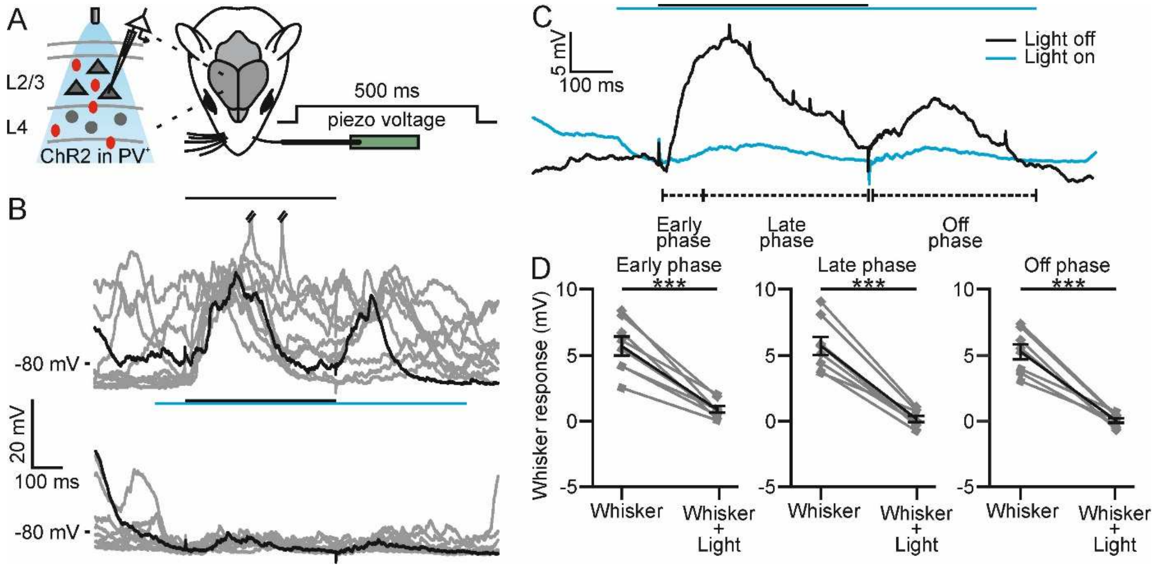

Supplementary Figure 4. Optogenetic activation of PV interneurons suppresses whisker-evoked responses in L2/3 principal neurons. (A) Schematic representation of the experimental configuration. Intracellular recordings in L2/3 pyramidal neurons were performed during whisker stimulation in anesthetized mice. Cortical activity was suppressed *via* optogenetic stimulation of ChR2-expressing PV-positive interneurons. Blue light (duration: 1 s) was delivered to S1bf through an optical fiber. Whisker stimulation was performed as described in Figure 2A. (B) Ten representative traces of an intracellular recording showing the membrane potential response of a L2/3 neuron during whisker stimulation in the absence (top) and presence (bottom) of optogenetic activation of PV cells (blue bar represents light illumination). AP spikes were truncated for presentation purposes. (C) Average of 30 traces showing the membrane potential response in the absence (black) and presence (blue) of optogenetic activation of PV interneurons for the cell shown in B. Early, late, and off phases are indicated below the traces (dashed lines). (D) Whisker-evoked depolarization in L2/3 pyramidal neurons during whisker stimulation in the absence (Whisker) and presence of optogenetic activation of PV interneurons (Whisker + Light) for the early (left), late (center), and off (right) phases. n = 8 cells from 3 animals; paired Student's *t*-test: p = 1E-4 for early phase; p = 3E-5 for late phase; p = 5E-5 for off phase, respectively.

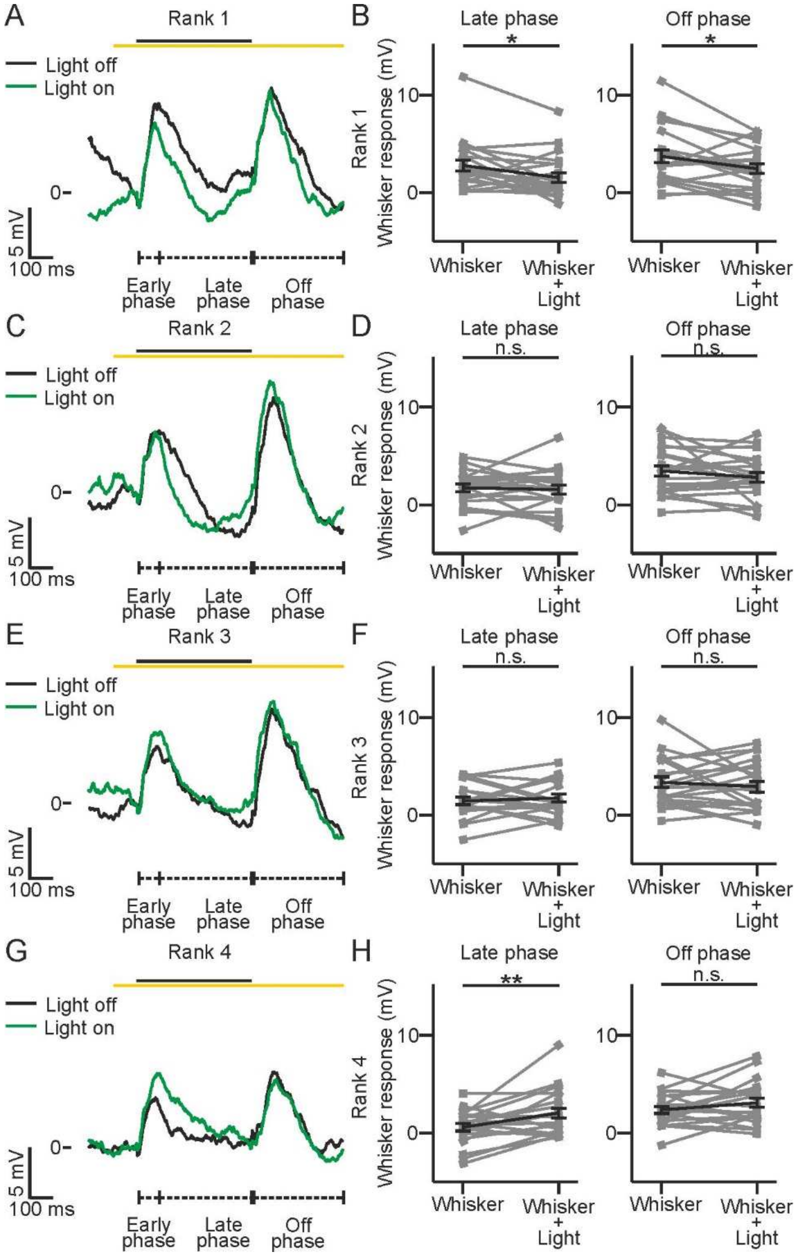

Supplementary Figure 5. Stimulus direction-specific control of L2/3 whisker response by L4 in anesthetized animals: late and off phases. (A) Same traces as in Figure 5B showing the average membrane potential of a L2/3 neuron in the absence (black) and presence (green) of L4 optogenetic inhibition during whisker stimulation in anesthetized mice for the Rank 1 angular direction. (B) Average amplitude of whisker-evoked responses for late (left) and off (right) phase in Rank 1 angular direction under the different experimental conditions. (C-D) Same as in A-B for Rank 2 angular direction. (E-F) Same as in A-B for Rank 3 angular direction. (G-H) Same as in A-B for Rank 4 angular direction. In B-D-F-H panels n = 21 cells from 12 animals. Rank 1: Wilcoxon paired signed rank test, p = 0.02 for late phase; paired Student's *t*-test, p = 0.012 for off phase. Rank 2: paired Student's *t*-test, p = 0.68 and 0.23 for late and off phase, respectively. Rank 3: paired Student's *t*-test, p = 0.50 and 0.42 for late and off phase, respectively. Rank 4: Wilcoxon paired signed rank test, p = 0.0096 for late phase; paired Student's *t*-test, p = 0.13 for off phase.

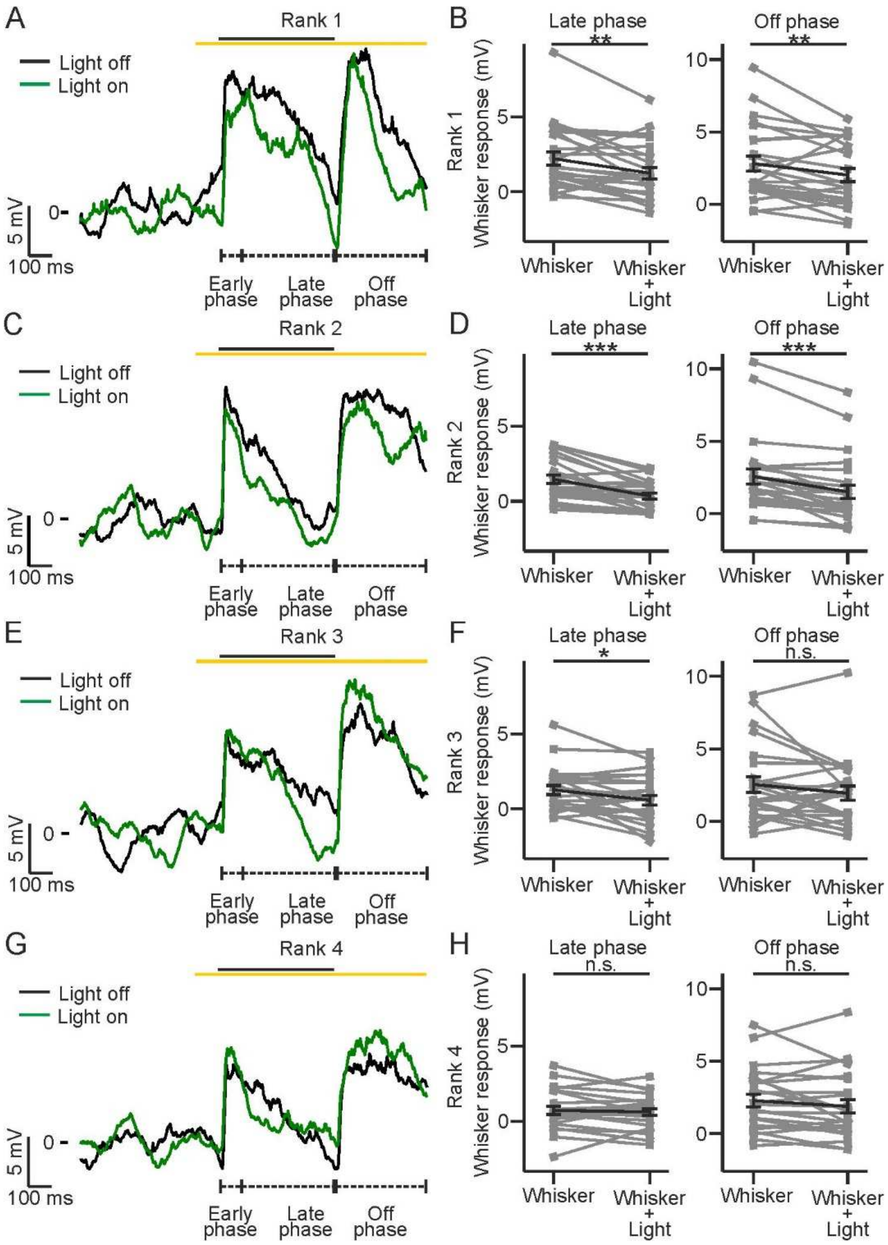

Supplementary Figure 6. Stimulus direction-specific control of L2/3 whisker response by L4 in awake animals: late and off phases. (A) Same traces as in Figure 6B showing the average membrane potential of a L2/3 neuron in the absence (black) and presence (green) of L4 optogenetic inhibition during whisker stimulation in awake mice for the Rank 1 angular direction. (B) Average amplitude of whisker-evoked responses for late (left) and off (right) phase in Rank 1 angular direction under the different experimental conditions. (C-D) Same as in A-B for Rank 2 angular direction. (E-F) Same as in A-B for Rank 3 angular direction. (G-H) Same as in A-B for Rank 4 angular direction. In B-D-F-H panels n = 24 cells from 11 animals. Rank 1: Wilcoxon paired signed rank test, p = 1.2E-3, 9E-3 for late and off phase, respectively. Rank 2: paired Student's *t*-test, p = 2E-5 for late phase; Wilcoxon paired signed rank test, p = 5E-5 for off phase. Rank 3: Wilcoxon paired signed rank test,

$p = 0.03$, 0.20 for late and off phase, respectively. Rank 4: paired Student's *t*-test, $p = 0.55$ for late phase; Wilcoxon paired signed rank test, $p = 0.12$ for off phase.
